# Giant Chylous Mesenteric Cyst Requiring Segmental Small Bowel Resection in a Young Adult: A Case Report

**DOI:** 10.1002/ccr3.73762

**Published:** 2026-10-19

**Authors:** Muhammad Wajid Siddique, Muhammad Hanzla Shahzad, Rabia Zameer, Hassaan Javaid, Elsa Obaid, Muhammad Abdullah Cheema, Nazila Dalir

**Affiliations:** ^1^ Ameer‐ud‐din Medical College Lahore Pakistan; ^2^ Larkin Community Hospital South Miami Florida USA; ^3^ Shifa College of Medicine Islamabad Pakistan; ^4^ Fatima Jinnah Medical University Lahore Pakistan; ^5^ School of Medicine St. George's University St. George's Grenada

**Keywords:** case report, chylous, histopathology, mesenteric cyst, Tillaux's sign

## Abstract

We report a 21‐year‐old male with a giant 20 × 20 × 15‐cm chylous mesenteric cyst. Such cysts present nonspecifically and pose diagnostic and surgical challenges. When a cyst is intimately attached to bowel and shares its mesenteric vascular supply, segmental bowel resection with complete cyst removal may be necessary because safe separation is not possible.

## 1 Introduction

A mesenteric cyst is a very rare benign intra‐abdominal mass occurring within the mesentery of the gastrointestinal tract. Symptoms are diverse, ranging from none to abdominal pain, abdominal swelling, and a palpable abdominal mass [1, 2, 3]. Intestinal obstruction, volvulus, bleeding, infection, and even rupture have also been reported as complications of these masses [1, 2, 3]. Because these cysts are rare and may present with a variety of symptoms, preoperative diagnosis can be challenging, as they are easily confused with other intra‐abdominal cystic masses [3, 4, 5].

The chylous mesenteric cyst is one of the rare types of these cystic masses and constitutes about 7%–9% of all mesenteric cysts. It is caused by developmental anomalies of the lymphatic system that lead to the accumulation of chyle in the cyst cavity [5, 6]. Because these cysts are lined by lymphatic endothelium, they are also described histologically as cystic lymphangiomas (lymphatic malformations) [2, 7]. In this report, “chylous mesenteric cyst” is used as the preferred term, and “lymphangioma type” refers to its histological classification rather than to a separate diagnosis. These cysts are rare in adults and tend to be asymptomatic until they reach a large size [2, 4, 8, 9]. Complete surgical resection remains the method of choice, but small bowel resection is sometimes required because of the relationship of the cyst to the bowel or the mesenteric vessels [3, 4, 5, 8].

Herein we describe a 21‐year‐old male with a giant chylous mesenteric cyst presenting with progressive abdominal pain, abdominal distension, and an enlarging abdominal mass. Tillaux's sign was positive, suggesting a mesenteric origin [10]. Complete excision required segmental small bowel resection because the cyst was intimately adherent to the bowel and shared its mesenteric vascular supply. Giant chylous mesenteric cysts in young adults have been reported before, including a recent case that was managed by complete enucleation without bowel resection [9]; the distinguishing feature of the present case is the shared vascular supply that made enucleation unsafe and necessitated bowel resection. This case highlights the diagnostic and operative challenges posed by giant chylous mesenteric cysts in young adults.

## 2 Case Presentation

A 21‐year‐old man presented with a one‐year history of progressive, vague, dull abdominal pain. This was accompanied by intermittent abdominal distension and a progressively enlarging palpable abdominal mass. There were no systemic or gastrointestinal symptoms such as nausea, vomiting, anorexia, fever, weight loss, or constipation.

He had no notable past medical or surgical history. Baseline investigations, including hemoglobin, serum electrolytes, liver function tests, renal function profile, and the tumor markers CEA and CA 19‐9, were all within normal reference ranges.

Physical examination showed an asymmetrically distended abdomen with a large, non‐tender, smooth, firm mass of approximately 20 × 20 cm occupying predominantly the central abdomen and extending toward the hypogastric region on palpation. The mass was considerably mobile when moved perpendicular to the axis of its mesenteric attachment, a finding known as Tillaux's sign. In this case, the positive sign suggested a mesenteric origin and was consistent with the subsequent imaging findings.

### 2.1 Investigations

Contrast‐enhanced CT of the abdomen and pelvis showed a massive, well‐defined, thin‐walled, multiloculated cystic mass measuring approximately 20 × 20 × 15 cm and arising from the small bowel mesentery, as shown in Figure 1. There was no calcification, fat attenuation, or solid mural component. The internal septa were thin, and the walls and septa showed slight enhancement after contrast administration. There was a pronounced mass effect, with lateral and superior displacement of adjacent loops of small bowel and colon. There was no evidence of mechanical bowel obstruction or local invasion.

**FIGURE 1:**
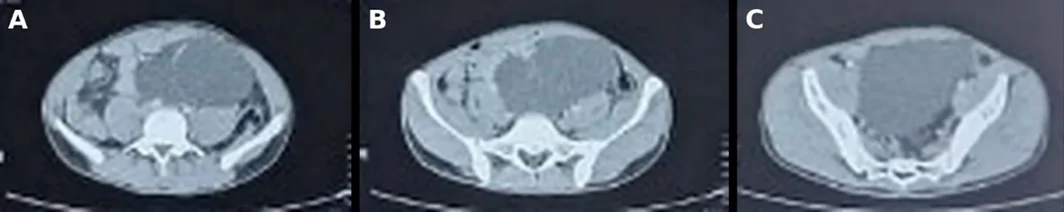
Axial contrast‐enhanced CT images of the abdomen and pelvis (A–C; representative slices from upper to lower levels) showing a giant, well‐defined, thin‐walled, multiloculated cystic mass of approximately 20 × 20 × 15 cm arising from the small bowel mesentery and displacing the adjacent small bowel loops and colon, without calcification or a solid mural component.

### 2.2 Management

Exploratory laparotomy was planned because of the large size of the mass, its progressive enlargement, and the patient's symptoms. At laparotomy, a giant multiloculated cyst originating from the small bowel mesentery was found.

The cyst was tightly adherent to the mesenteric border of a segment of small bowel and shared the mesenteric vascular supply of that segment (Figure 2). Enucleation would have required dividing or injuring the vessels supplying the involved bowel, with a risk of bowel ischemia or injury to the bowel wall, so safe separation of the cyst from the bowel was not possible. A segmental resection of the involved small bowel, 18 cm in length, was therefore performed together with the entire cyst (Figure 3).

**FIGURE 2:**
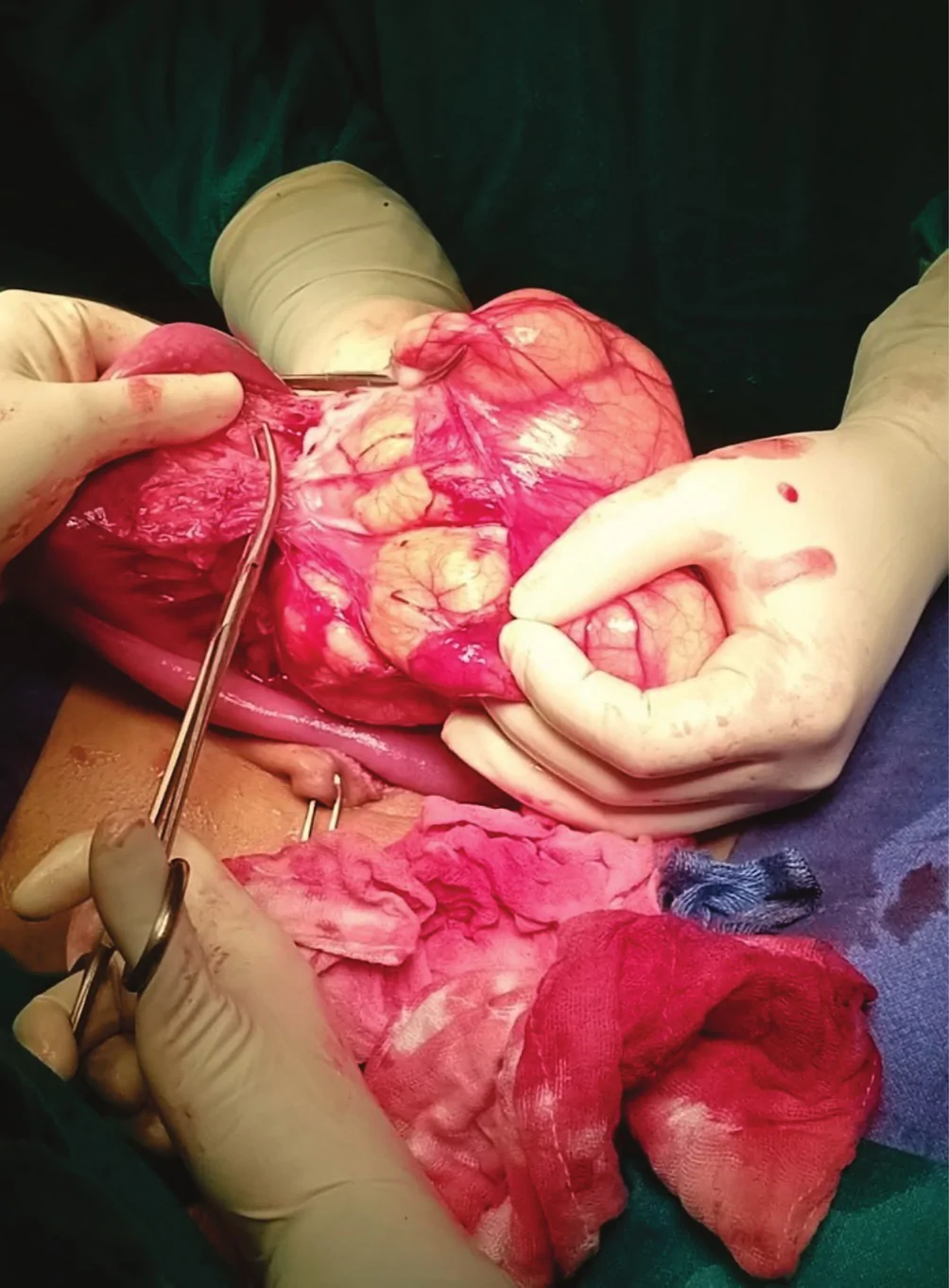
Intraoperative photograph showing the giant multiloculated, thin‐walled cyst with visible surface vessels, tightly adherent to the mesenteric border of the adjacent small bowel loop and sharing its mesenteric vascular supply, which made safe enucleation impossible.

**FIGURE 3:**
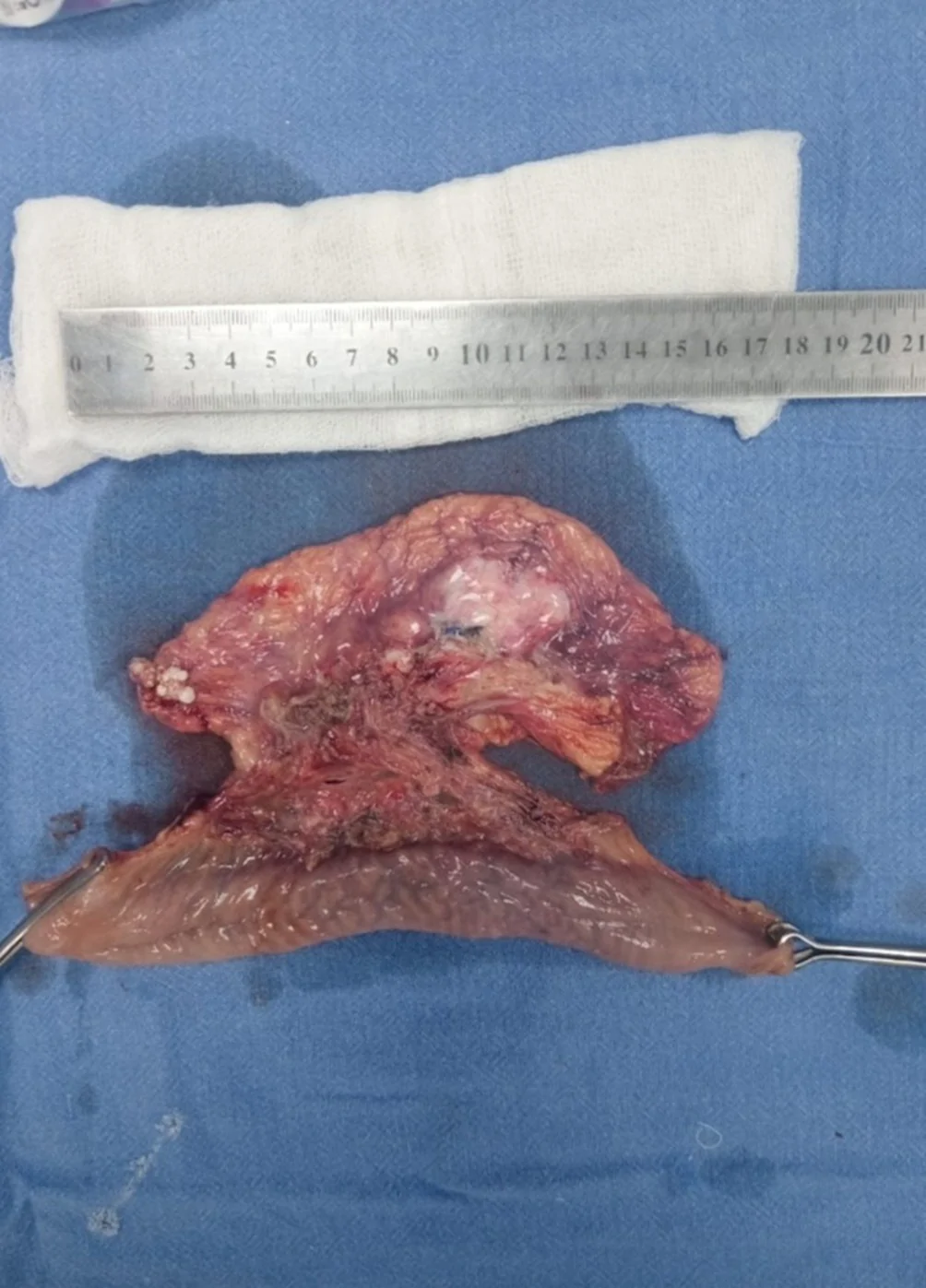
Resected specimen: the segment of small bowel, opened to show the mucosal surface, with the attached collapsed multiloculated cyst wall along its mesenteric border after decompression of the chylous contents. Scale in centimeters.

During the operation, a controlled incision into one locule released a copious amount of characteristic milky chylous fluid, which decompressed the cyst and indicated a lymphatic origin. Intestinal continuity was restored by a primary anastomosis between the two cut ends of the small bowel.

### 2.3 Histopathology and Immunohistochemistry

Grossly, the specimen consisted of an 11‐cm segment of small bowel, approximately 2 cm in circumference, that was tightly adherent to a multiloculated mesenteric cyst measuring 12 × 7 cm, with walls up to 2 mm thick and no papillary or solid components on the external surface. These dimensions are smaller than those recorded on CT and at surgery (a cyst of 20 × 20 × 15 cm and an 18‐cm bowel segment) because the specimen was measured after the cyst had collapsed following drainage of its chylous contents and after the resected bowel segment had contracted; the pathological measurements therefore describe the collapsed specimen rather than the in situ dimensions.

Microscopically, the cyst wall showed a uniform architecture and consisted of a very thin layer of fibrocollagenous tissue containing minimal chronic inflammatory infiltrate and a few scattered smooth muscle fibers. This connective tissue layer was lined by a single layer of bland cuboidal to flattened (attenuated) cells without atypia or other worrisome histological features. The adjacent small bowel was architecturally normal, with well‐developed, regular villi covered by tall columnar epithelium and only minimal nonspecific chronic inflammation in the lamina propria; there were no granulomas, atypia, or malignancy.

Immunohistochemistry was performed to distinguish a lymphatic cyst from other cystic lesions of the mesentery, namely mesothelial, enteric duplication, and Müllerian cysts. The lining cells showed strong positivity for the lymphatic endothelial markers D2‐40 (podoplanin) and CD31. They were negative for the mesothelial markers calretinin and WT‐1, for the epithelial marker pan‐cytokeratin, and for the hormone receptors ER and PR, which excluded mesothelial, epithelial, and Müllerian cysts.

Together, the histological features, the intraoperative finding of chylous fluid, and the immunohistochemical profile established the final diagnosis of a benign chylous mesenteric cyst (lymphangioma type) of the small bowel mesentery.

### 2.4 Postoperative Outcomes and Follow‐Up

The postoperative course was uneventful. The patient resumed oral intake on postoperative Day 3, passed flatus and stool, and was discharged in stable condition on postoperative Day 6. At the 6‐month follow‐up, his abdominal pain and distension had completely resolved. There was no palpable intra‐abdominal mass or localized tenderness on clinical examination. Surveillance abdominal ultrasound showed no abnormality, with normal‐appearing bowel loops and no fluid collection or cystic lesion. Thus, at 6 months he remained asymptomatic with no ultrasonographic evidence of recurrence; longer follow‐up is needed to assess the long‐term outcome.

### 2.5 Summary of Clinical Events

A summary of the clinical events is presented in Table 1.

**TABLE 1:** Summary of clinical events.

Time	Clinical event
1 year	Progressive abdominal pain and distension
Presentation	20 × 20 cm abdominal mass; positive Tillaux's sign
CT	20 × 20 × 15 cm mesenteric cyst
Surgery	Cyst excision +18‐cm small‐bowel resection and anastomosis
Histopathology	Chylous mesenteric cyst confirmed
Day 6	Discharged stable
6 months	Asymptomatic; no recurrence on ultrasound

## 3 Discussion

### 3.1 Epidemiology and Clinical Presentation

Mesenteric cysts are rare benign intra‐abdominal lesions, occurring in approximately 1 in 100,000 adult hospital admissions. Chylous mesenteric cysts are a rare subgroup arising mostly in the small intestinal mesentery; they are thought to result from congenital anomalies of lymphatic vessels that do not communicate with the lymphatic system, and lymph accumulation causes progressive enlargement and mass effect [1, 2, 3, 7]. Presentation is nonspecific, ranging from incidental asymptomatic lesions to pain, distension, early satiety, or a palpable mass and, rarely, complications such as obstruction, volvulus, hemorrhage, rupture, or infection, so diagnosis is often delayed [2, 5]. Our patient's one‐year course of progressive pain and distension without systemic symptoms is typical of giant cysts diagnosed only after considerable growth [2, 7].

The positive Tillaux's sign, which is not commonly highlighted in modern literature, suggested a mesenteric origin in our patient and was consistent with the subsequent imaging findings [6].

### 3.2 Role of Imaging

Contrast‐enhanced CT is the preferred imaging technique: it delineates the size and internal features of the cyst and its relationship to adjacent bowel and vessels and helps to exclude malignancy. Typical features are a well‐defined, thin‐walled, septated cystic lesion without solid components [3, 5]. In our patient, CT showed a 20 × 20 × 15 cm multiloculated cyst with thin enhancing septa and displaced bowel loops, without mural nodules, calcification, or infiltration, which suggested a benign lesion. However, imaging alone cannot reliably distinguish chylous mesenteric cysts from duplication cysts, mesothelial cysts, pseudocysts, or cystic neoplasms, so histopathology remains essential for diagnosis [2, 5].

### 3.3 Surgical Management and Distinguishing Features

Complete excision is the treatment of choice for mesenteric cysts because incomplete removal increases the risk of recurrence, and aspiration or marsupialization has been largely abandoned [1, 3, 7]. Enucleation with preservation of the bowel is preferable when feasible, as in a recent giant chylous mesenteric cyst in a young adult that was enucleated without bowel resection [9].

In our patient, enucleation was not possible because the cyst was intimately adherent to the small bowel and shared its mesenteric vascular supply; attempted separation risked vascular injury and bowel ischemia, so segmental resection with primary anastomosis was performed. Bowel resection has similarly been reported when the mesenteric vessels are involved or the cyst cannot be separated from the bowel [2, 11], suggesting that it may be necessary when safe separation is not possible.

This distinguishes the present case from previously reported giant chylous mesenteric cysts in young adults, including that of Tiruneh et al. [9]: the intimate relationship of the cyst with the small bowel mesenteric vascular supply precluded safe enucleation and required bowel resection. The practical lesson is to assess the relationship between the cyst, bowel, and mesenteric vessels early and to be prepared for resection if safe separation is not possible.

### 3.4 Histopathological Findings

Histopathology remains the gold standard for diagnosis. In our patient, a thin fibrocollagenous cyst wall lined by bland cuboidal to flattened cells that were positive for D2‐40 and CD31 and negative for calretinin, WT‐1, cytokeratin, and ER/PR, together with the chylous fluid found at operation, established a benign chylous mesenteric cyst (lymphangioma type) and excluded mesothelial, epithelial, and Müllerian cysts [11, 12].

### 3.5 Outcome and Clinical Implications

Complete excision is generally associated with symptomatic relief and low recurrence rates [1, 3]. Our patient recovered well and was discharged on postoperative Day 6; at 6 months he was asymptomatic without ultrasonographic recurrence, in keeping with previous reports [3, 7]. However, 6 months is a short follow‐up period, and longer surveillance is needed to confirm a durable result.

As a single case with short follow‐up, our findings cannot establish bowel resection as the standard of care. Nevertheless, when a cyst shares the vascular supply of the adjacent bowel, resection of that bowel segment may be a means of achieving complete excision rather than a complication in itself.

## 4 Conclusion

This case demonstrates that a mesenteric cyst may remain asymptomatic or present with nonspecific symptoms for an extended period before becoming clinically apparent. When the cyst shares the mesenteric vascular supply of the adjacent small bowel, simple enucleation may not be safe, and segmental resection of the involved bowel with primary anastomosis may be necessary to achieve complete excision. The uneventful postoperative course and the absence of recurrence at 6 months are encouraging, but longer follow‐up is needed to confirm the outcome.

## Author Contributions

**Muhammad Wajid Siddique:** conceptualization, writing – original draft, methodology, validation, visualization, writing – review and editing, project administration, data curation, supervision, resources. **Muhammad Hanzla Shahzad:** supervision, writing – original draft, writing – review and editing. **Rabia Zameer:** writing – original draft. **Hassaan Javaid:** writing – original draft. **Elsa Obaid:** writing – original draft. **Muhammad Abdullah Cheema:** writing – original draft. **Nazila Dalir:** writing – review and editing.

## Funding

The authors have nothing to report.

## Ethics Statement

This case report was conducted in accordance with the principles of the Declaration of Helsinki. Written informed consent was obtained from the patient for publication of this case report and any accompanying images. No institutional ethics committee approval was required for this individual case report; however, patient confidentiality and anonymity were maintained throughout.

## Consent

Written informed consent was obtained from the patient for publication of this case report, including all clinical details and images, in accordance with the journal's policies.

## Conflicts of Interest

The authors declare no conflicts of interest.

## Data Availability

The data that support the findings of this case report are available from the corresponding author upon reasonable request.
